# Sale of Private Equity–Owned Physician Practices and Physician Turnover

**DOI:** 10.1001/jamahealthforum.2024.5376

**Published:** 2025-02-14

**Authors:** Victoria Berquist, Lev Klarnet, Leemore Dafny

**Affiliations:** 1 Harvard Kennedy School, Harvard University, Cambridge, Massachusetts; 2 Harvard Business School, Harvard University, Boston, Massachusetts; 3 Department of Economics, Harvard University, Cambridge, Massachusetts; 4 National Bureau of Economic Research, Cambridge, Massachusetts

## Abstract

**Importance:**

Private equity (PE) acquisition of physician practices is increasing, with owners targeting sales, or *exits*, in 3 to 7 years. Little is known about the association of exit with physician retention and subsequent employment.

**Objective:**

To examine whether PE exit of physician practices is associated with changes in physician retention and subsequent choice of practice size.

**Design, Setting, and Participants:**

Using data from the Centers for Medicare & Medicaid Services Doctors and Clinicians National Downloadable File from December 31, 2014, to December 31, 2020, this case-control study compared employment changes for physicians at PE-exiting practices sold between January 1, 2016, and December 31, 2018 (treatment group), with employment changes for matched control physicians in practices not sold by PE owners but with the same specialty, hospital referral region, practice size, and time period. Physicians billing fee-for-service Medicare during the study period were eligible for inclusion. A difference-in-differences design was used to compare retention between the treatment and control groups in the 2 years before and after exit using a multinomial logit model that adjusted for physician decade of graduation. Data were analyzed from August 1, 2023, to November 9, 2024.

**Exposure:**

Exit of a PE-owned physician practice.

**Main Outcomes and Measures:**

Physician employment outcomes included staying (continuing to bill through the initial practice), working elsewhere (only billing through other practices), and retirement (no longer billing). Whether a physician left to a join large (>120-physician) practice was also evaluated.

**Results:**

Of the 1215 physicians included in the analysis (405 at 70 PE-exiting practices and 810 matched controls; 814 [67.0%] male and 401 (33.0%) were female. Physicians in all PE-exiting practices were typically in practices of more than 20 physicians (471 [65.2%]) and often in the South (373 [51.7%]). Dermatology was the leading specialty (216 [29.9%]), followed by family medicine (94 [13.0%]). Physicians employed in PE-exiting practices were 16.5 (95% CI, 10.6-22.3) percentage points less likely to continue working in that practice 2 years after exit compared with matched controls. There was no significant change in the probability of retirement (0 percentage points; 95% CI, −4.1 to 4.0). Physicians in PE-exiting practices were 10.1 (95% CI, 6.5 to 13.7) percentage points likelier than matched controls to join a large practice of more than 120 physicians.

**Conclusions and Relevance:**

In this case-control study, PE exit was followed by an increase in physician turnover and subsequent employment at a large (>120-physician) practice relative to matched controls, notwithstanding similar turnover rates between these physicians and matched controls prior to exit. The increase in physician turnover and consolidation following PE exits has important implications for patients, physicians, investors, and physician markets.

## Key Points

**Question:**

What is the association between the sale of private equity (PE)–owned physician practices and subsequent physician turnover and employment decisions?

**Findings:**

In this case-control study of 1215 physicians, those in practices sold by PE owners were 16.5 percentage points likelier to work elsewhere within 2 years after the sale and 10.1 percentage points likelier to join large (>120-physician) practices upon leaving than matched control physicians working in the same local markets and specialties in practices not sold by PE owners.

**Meaning:**

The increase in physician turnover and consolidation following PE exits has important implications for patients, physicians, investors, and physician markets, including disruption for practices and patients and likely for increases in costs of care.

## Teaser

This case-control study using data from the Centers for Medicare & Medicaid Services Doctors and Clinicians National Downloadable File compares changes in physician employment in private equity–exiting practices with those of physicians who did not work in these practices during the same period.

## Introduction

Private equity (PE) has become increasingly important in US health care over the last 20 years. Over 800 PE health care transactions were completed in 2022; total deal value over the preceding decade is estimated at $750 billion.^1,2^ Physician practices have become an attractive target: between 2010 and 2020, PE investors purchased over 1000 US practices.^3^ Typically, PE investors seek a return within 3 to 7 years.^4^ Some stakeholders have raised concerns that these short-term objectives may conflict with the longer-term interests of patients, payers, and physicians.^5,6^

Several prior studies examined the effects of PE acquisitions of physician practices.^7,8,9,10^ These document postacquisition increases in charges, allowed amounts per claim, encounter volumes, and new patient volume.^7,8,9^ One study examining the effects on clinicians found that PE acquisition was followed by increased turnover and a shift toward advanced practice clinicians.^10^ However, what happens after PE exits from physician practice investments (ie, the PE investor sells the practice) remains unexplored.

Exits may impact physician retention for several reasons, including changes in financial incentives to continue working in the practice. Many PE-acquired practices were previously independent, run by physician-owners and acquired for substantial sums, reportedly 3 to 13 times practice earnings.^11,12^ PE acquisitions are typically structured to provide financial incentives for physician-owners to stay for a period, but these incentives may disappear after the initial PE investor exits the practice.^12,13^ However, the exiting PE firm may incentivize anchor physicians to reroll their equity to reassure buyers they will stay after purchase.^14,15^ Ultimately, the effect of PE exit on physician retention is an empirical question.

An impact on retention, if present, may have important implications for patients, physicians, investors, and physician markets. Turnover may disrupt patient-physician relationships, with potentially adverse effects on patient outcomes,^16,17^ and it may also reflect negative physician experiences with PE owners. Investors may want to incorporate expectations regarding retention into their strategies. Finally, accelerated post–PE exit departures may contribute to broader shifts away from independent practices toward larger hospital systems and corporations. In this study, we examine physician employment decisions following the exit of initial PE investors from physician practices.

## Methods

In this case-control study, we assessed the association between PE exits and physician employment decisions by comparing changes in employment for physicians who worked in PE-exiting practices (treatment group) with changes in employment during the same period for matched physicians who did not work in PE-exiting practices (control group). We studied employment changes from 2 years before to 2 years after exit. Outcomes included staying (continue billing from the same practice), working elsewhere (stop billing from the same practice but continue billing from another), and retirement (no longer observed to be practicing in our data). Institutional review board approval was not sought because, based on guidance from the Harvard Business School, this study did not constitute human subjects research. This study aligns with the Strengthening the Reporting of Observational Studies in Epidemiology (STROBE) reporting guideline.

### Data Sources

PE exits of physician practices were identified using the PitchBook data platform (PitchBook Inc) and manually verified using internet searches for sale evidence (the eMethods in Supplement 1 provides additional details). An exit was defined as the sale of a practice or group of practices by a PE firm; PE firms may “roll up” or acquire multiple practices and then sell them as a consolidated company, and therefore 1 exit may involve several practices. We focus exclusively on the first observed exit for a PE-owned practice or practice group.

Physician-level data were obtained from the Centers for Medicare & Medicaid Services Doctors and Clinicians National Downloadable File (NDF) from December 31, 2014, to December 31, 2020, which spans at least 2 years before and 2 years after each PE exit.

The NDF includes all physicians who billed a fee-for-service claim to Medicare in the prior 6 months. We used the December or latest file available each year. Practices in the NDF were matched to PE exits using practice names and addresses.

Physicians may be affiliated with more than 1 practice. Physicians were assigned to the treatment group if they billed Medicare from any PE-exiting practice in the year before exit. When evaluating employment destinations, a physician who continued to bill from the same PE-exiting practice in subsequent years was classified as staying even if that physician also billed through other practices.

### Statistical Analysis

Data were analyzed from August 1, 2023, to November 9, 2024. To form a control group, we matched each physician working in a PE-exited practice in the year before exit to 2 physicians in practices that did not undergo a PE exit. Practices with over 120 clinicians, reflecting the maximum size of PE-exiting practices in our sample, were eliminated from the set of potential controls. Larger groups are more likely to be affiliated with hospitals and may therefore be less comparable to the types of practices acquired and sold by PE firms. Because the control group was not restricted by ownership type, some members may have been employed in PE-owned practices.

Treatment physicians were matched with controls in the same specialty, hospital referral region (HRR), and practice size (<5, 5-19, and 20-120 physicians). Job opportunities and therefore retention can be expected to vary by specialty and geography. Practice size was used as a matching factor because prior evidence suggests that turnover may be higher in larger practices.^18^ Matches were selected without replacement using exact field matching; in cases with more than 2 exact matches, 2 were selected at random.

We used unpaired *t* tests comparing mean employment outcomes for treatment and control physicians in each year relative to PE exit and multinomial logit regressions to analyze all study years collectively. The multinomial logit used a difference-in-differences specification to estimate the association between PE exit and employment decisions for each year relative to exit. These regressions controlled for time trends using year-relative-to-exit fixed effects and adjusted for the association of age with retirement by controlling for graduation decade (1980 or earlier, 1981-1990, 1991-2000, and 2001 or later).

For robustness, we evaluated results in 4 alternative samples. Two of these varied the matching approach, with the first alternative limited by requiring 5 matches for each physician in the treatment group and the second alternative expanded by relaxing the geographic match criterion to the Census division. Two additional alternative samples implemented different, lower ceilings for practice size among matched controls (see the eMethods in Supplement 1 for details). We also examined the heterogeneity of employment responses to PE exit by specialty, graduation year, and whether the buyer was a PE firm using interactions between these characteristics and the indicator for PE exit (see the eMethods in Supplement 1 for details and additional robustness checks).

Coefficients from the multinomial logit regression analyses are reported as mean marginal effects and can be interpreted as percentage point changes in the probability of each outcome associated with the relevant indicator variable. Statistical significance was determined as 2-sided *P* < .05. SEs were clustered at the physician level; robustness to clustering at the practice level was also evaluated. All analyses were conducted using Stata, release 18.0 (StataCorp LLC).

## Results

We identified 63 PE exits between 2016 and 2018. Of these, 52 (82.5%) corresponded to 1 or more practices observed in the NDF, yielding 74 unique practices. Of the 722 physicians affiliated with the 74 practices in the year prior to exit, we were able to identify 2 control physicians for 405 physicians affiliated with 70 practices; these physicians and their matches constitute our primary analysis sample. Thus, a total of 1215 physicians were included in the analysis, of whom 814 (67.0%) were male and 401 (33.0%) were female. Table 1 presents descriptive statistics separately for all PE-exiting physicians, the subset for which we could identify 2 matched controls (ie, the 405 in the treatment group), and matched controls (ie, the 810 in the control group). Physicians in all PE-exiting practices were typically in practices of more than 20 physicians (471 [65.2%]) and often in the South (373 [51.7%]). Dermatology was the leading specialty (216 [29.9%]), followed by family medicine (94 [13.0%]). The distribution of physicians across graduation decade was similar for the treatment and control groups. Characteristics of practices included in the primary analysis sample were also similar to those of all PE-exiting practices (see eTable 1 in Supplement 1).

**Table 1.** Balance Table Comparing PE-Exiting Physicians With Matched Controls^a^

Characteristic	Physicians, No. (%)			
	All PE-exited physicians (n = 722 in 74 practices)	Primary sample		Difference (95% CI), percentage points
		PE-exited physicians (n = 405 in 70 practices)	Matched physicians (n = 810 in 396 practices)	
No. of physicians in practice				
<5	71 (9.8)	64 (15.8)	128 (15.8)	0 (−4.4 to 4.4)
5-19	180 (24.9)	129 (31.9)	258 (31.9)	0 (−5.6 to 5.6)
≥20	471 (65.2)	212 (52.3)	424 (52.3)	0 (−6.0 to 6.0)
Region				
South	373 (51.7)	237 (58.5)	474 (58.5)	0 (−5.9 to 5.9)
Northeast	100 (13.9)	70 (17.3)	139 (17.2)	0.1 (−4.4 to 4.6)
Midwest	216 (29.9)	66 (16.3)	133 (16.4)	−0.1 (−4.5 to 4.3)
West	33 (4.6)	32 (7.9)	64 (7.9)	0 (−3.2 to 3.2)
Year of graduation				
1980 or Earlier	131 (18.1)	59 (14.6)	149 (18.4)	−3.8 (−8.3 to 0.7)
1981-1990	149 (20.6)	84 (20.7)	175 (21.6)	−0.9 (−5.8 to 4.0)
1991-2000	203 (28.1)	123 (30.4)	220 (27.2)	3.2 (−2.2 to 8.6)
2001 or Later	239 (33.1)	139 (34.3)	266 (32.8)	1.5 (−4.1 to 7.1)
Specialty				
Dermatology	216 (29.9)	94 (23.2)	188 (23.2)	0 (−5.0 to 5.0)
Family medicine	94 (13.0)	94 (23.2)	188 (23.2)	0 (−5.0 to 5.0)
Ophthalmology	55 (7.6)	44 (10.9)	88 (10.9)	0 (−3.7 to 3.7)
Anesthesiology	37 (5.1)	37 (9.1)	74 (9.1)	0 (−3.4 to 3.4)
Internal medicine	34 (4.7)	34 (8.4)	68 (8.4)	0 (−3.3 to 3.3)
Emergency medicine	31 (4.3)	26 (6.4)	52 (6.4)	0 (−2.9 to 2.9)
General surgery	9 (1.2)	9 (2.2)	18 (2.2)	0 (−1.8 to 1.8)
Radiology	12 (1.7)	8 (2.0)	16 (2.0)	0 (−1.7 to 1.7)
Pain management	75 (10.4)	7 (1.7)	14 (1.7)	0 (−1.6 to 1.6)
Urology	95 (13.2)	7 (1.7)	14 (1.7)	0 (−1.6 to 1.6)
Nephrology	11 (1.5)	5 (1.2)	10 (1.2)	0 (−1.3 to 1.3)
Other	53 (7.3)	40 (9.9)	80 (9.9)	0 (−3.6 to 3.6)

Abbreviation: PE, private equity.

^a^ All PE-exiting physicians were identified in the Doctors and Clinicians National Downloadable File. Controls were selected using exact matching by practice size, specialty, and hospital referral region. Nonzero differences across regions for the treatment and control groups occur because hospital referral regions occasionally span more than 1 region. Not all physicians from a practice are necessarily included due to matching limitations. All variables are measured in the year prior to exit (ie, 2015, 2016, or 2017).

### Association of PE Exit With Physician Employment Decisions

**Figure 1.**
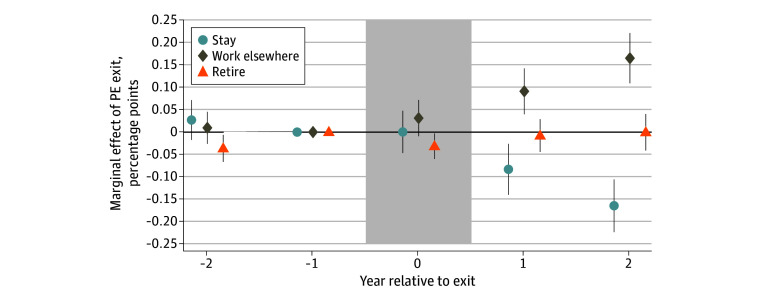
Employment Decisions of Physicians in Private Equity (PE)–Exiting Practices Relative to Controls, Before and After PE Exit The graph plots the coefficients from the multinomial logit specification for physician employment decisions for the primary sample of 405 treatment physicians and 810 control physicians. All coefficients are estimated in a single pooled regression controlling for graduation decade and year relative to exit (year 0). The points represent the corresponding coefficients, and the vertical bars are 95% CIs. eTable 2 in Supplement 1 reports the tabular results underlying this figure, as well as coefficients and 95% CIs for the control variables. The data are at the physician-year level, and coefficient estimates for staying and retirement are offset on the x-axis slightly for readability. The shaded region represents the year of PE exit. SEs are clustered at the physician level.

The results of the multinomial logit regression (Figure 1) show physicians employed in practices experiencing PE exits were 16.5 (95% CI, 10.6-22.3) percentage points less likely to continue working in that practice 2 years after exit than matched controls. There was no significant change in retirement probability (0 percentage points; 95% CI, −4.1 to 4.0). Instead, physicians were 16.5 (95% CI, 10.9-22.1) percentage points more likely to practice elsewhere. A comparison of unadjusted means yielded similar results (Table 2).

**Table 2.** Physician Employment Decisions 1 and 2 Years After PE Exit

Employment outcome	Physicans, No. (%)		Difference (95% CI), percentage points^a^
	PE-exited physicians (n = 405)	Matched physicians (n = 810)	
**1 y After exit**			
Stayed	246 (60.7)	560 (69.1)	−8.4 (−14.0 to −2.8)
Retired	41 (10.1)	91 (11.2)	−1.1 (−4.8 to 2.6)
Worked elsewhere	118 (29.1)	159 (19.6)	9.5 (4.5 to 14.5)
Left to large practice^b^	46 (11.4)	62 (7.7)	3.7 (0.3 to 7.1)
Left to small practice^c^	72 (17.8)	97 (12.0)	5.8 (1.7 to 9.9)
**2 y After exit**			
Stayed	176 (43.5)	486 (60.0)	−16.5 (−22.4 to −10.7)
Retired	55 (13.6)	114 (14.1)	−0.5 (−4.6 to 3.6)
Worked elsewhere	174 (43.0)	210 (25.9)	17.0 (11.6 to 22.5)
Left to large practice^b^	70 (17.3)	58 (7.2)	10.1 (6.5 to 13.7)
Left to small practice^c^	104 (25.7)	152 (18.8)	6.9 (2.1 to 11.8)

Abbreviation: PE, private equity.

^a^ Defined as the difference in unadjusted means between PE-exited physicians and matched physicians.

^b^ Defined as working elsewhere (ie, no longer observed at the original practice prior to PE exit) and observed at a practice with over 120 physicians.

^c^ Defined as working elsewhere and observed only at practices with 120 or fewer physicians.

Figure 1 shows that there were no statistically significant differences in physicians’ probability of staying or working elsewhere between the treatment and control groups in the 2 years prior to exit, suggesting that turnover among control physicians is a reasonable counterfactual for turnover among treatment physicians absent PE exit. We observed slightly lower propensities of retirement among physicians in PE-exiting practices 2 years prior to exit (−3.6 percentage points; 95% CI −6.6 to −0.6). Analyses examining the presence of heterogenous effects by physician cohort show that this result is due to relatively younger physicians (graduation year after 1990) in the treatment group (see eFigure 1 in Supplement 1). Focusing on physicians who graduated in 1990 or earlier—the group more plausibly induced to retire after PE exit—there was no statistically significant difference between treatment and control groups in the probability of retirement 2 years before exit (0.3 percentage points; 95% CI, −7.0 to 7.6) or 2 years after exit (2.3 percentage points; 95% CI, −4.0 to 8.6).

Results obtained using the alternative samples of physicians were similar (see eFigures 2, 3, 5, and 6 in Supplement 1). The statistical significance of the results was minimally impacted by clustering SEs at the practice level (eFigure 4 in Supplement 1).

### Heterogeneity by Buyer Type and Physician Characteristics

**Figure 2.**
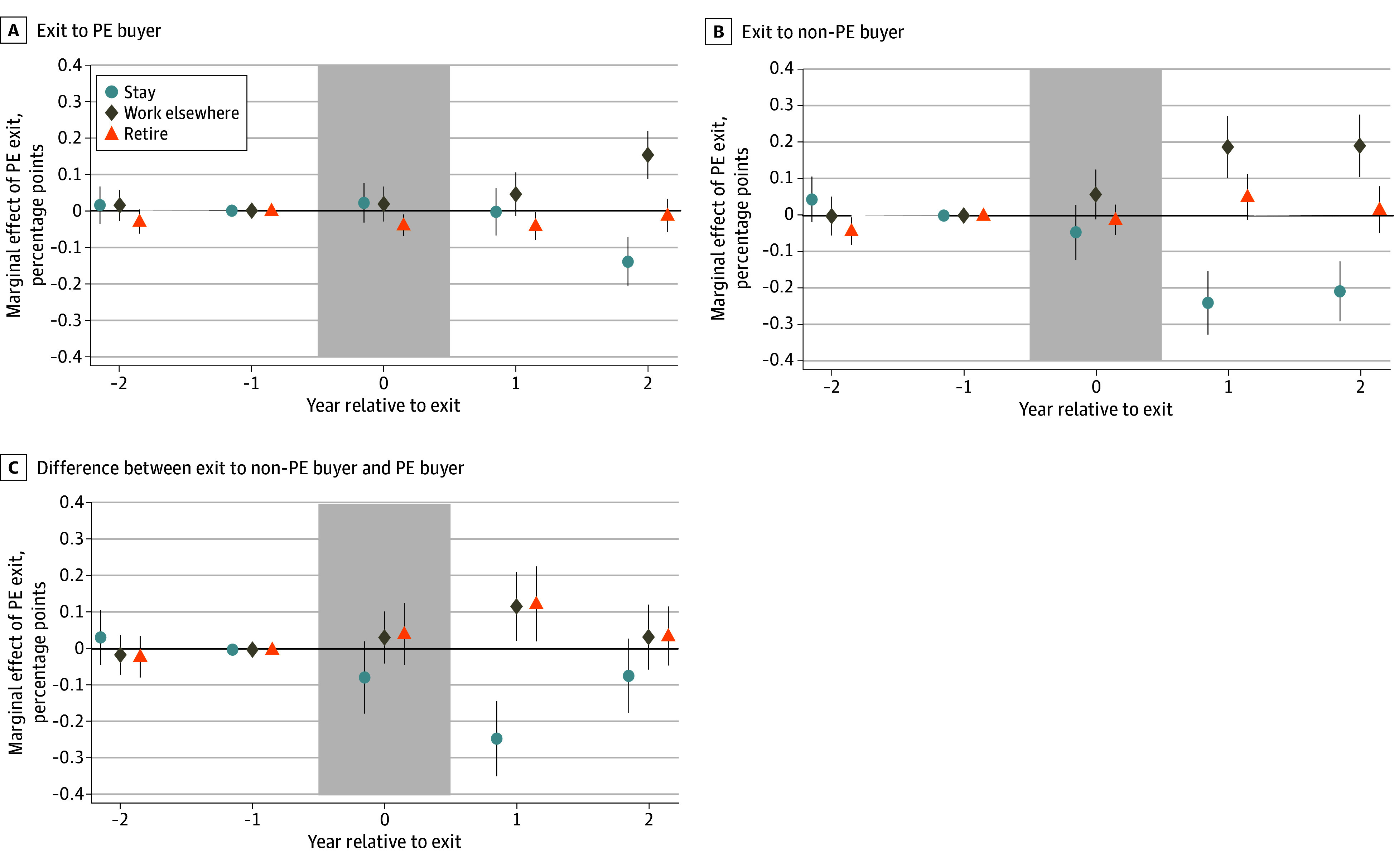
Employment Decisions of Physicians in Private Equity (PE)–Exiting Practices Relative to Controls, Before and After PE Exit, by Buyer Type The graphs plot the coefficients from multinomial logit specifications for physician employment decisions in which the indicators for years relative to PE exit are interacted with indicators reflecting whether the practice was purchased by a PE or non-PE buyer. Practices of 265 of the 405 treatment physicians were sold to PE buyers. The data are at the physician-year level and coefficient estimates for staying and retirement are offset on the x-axis slightly for readability. The points represent the corresponding coefficients, and the vertical bars are 95% CIs. A and B, Data are estimated using a single, pooled multinomial logit regression where a PE exit indicator for each year relative to exit is interacted separately with indicators for PE buyer and non-PE buyer. C, Plot of the interaction term of a single, pooled multinomial logit regression with an indicator for PE exit in each year relative to exit and an indicator for PE exit in each year relative to exit interacted with an indicator for non-PE buyer. Both regressions control for graduation year decade and year relative to exit. The shaded region represents the year of PE exit. SEs are clustered at the physician level.

Fifty-two of the 70 PE-exiting practices in our primary sample were sold to PE buyers, which represented 265 of the 405 treatment physicians. High share of PE-to-PE sales of physician practices was recently documented by Singh et al.^19^ Figure 2 shows that while physicians in PE-owned practices sold to non-PE buyers were significantly more likely to work elsewhere within the first year after exit, by 2 years after exit, turnover at practices sold to PE buyers had accelerated and the difference was no longer statistically significant.

The probability of staying after PE exit was lower in family medicine by 14.7 (95% CI, 0.0-29.3) percentage points and dermatology by 14.1 (95% CI, −0.1 to 28.3) percentage points relative to other specialties (eFigure 7 in Supplement 1). There were no statistically significant differences in postexit employment decisions for older vs younger physicians in the treatment group relative to controls (eFigure 1 in Supplement 1).

### Association of PE Exit With Concentration

To evaluate whether PE exits result in more physicians joining large (likely hospital- or corporate-owned) practices rather than returning to smaller practices (ie, typical practice sizes before PE acquisition), we compared differences between the treatment and control groups in the share of physicians leaving to large (>120-physician) vs small (≤120-physician) practices. Physicians affiliated with multiple practices following exit were assigned to their largest practice observed each year.

Table 2 shows that physicians in PE-exiting practices were 10.1 (95% CI, 6.5-13.7) percentage points more likely than matched controls to leave to a large practice 2 years after PE exit. This difference can be decomposed into 2 factors. First, as shown in Table 2, PE-exiting physicians were 17.0 (95% CI, 11.6-22.5) percentage points more likely than controls to leave their practice to work elsewhere 2 years after exit. Second, as shown in Table 3, conditional on leaving within 2 years of exit, treatment physicians were 12.6 (95% CI, 3.2-22.0) percentage points more likely to join a large practice than controls. The greater propensity for PE-exiting physicians to join large practices conditional on leaving was not present for physicians working in those practices 2 years prior to exit, suggesting that this result cannot be explained by a preference for large practices among treatment physicians relative to controls prior to exit (eTable 3 in Supplement 1).

**Table 3.** Physician Employment Decisions Among Physicians Leaving to Work Elsewhere After PE Exit

Employment outcome conditional on working elsewhere	Physicians, No./total No. (%)		Difference (95% CI), percentage points
	PE-exited physicians	Matched physicians	
**1 y After exit**			
Left to large practice^a^	46/118 (39.0)	62/159 (39.0)	0 (−11.7 to 11.6)
Left to small practice^b^	72/118 (61.0)	97/159 (61.0)	0 (−11.6 to 11.7)
**2 y After exit**			
Left to large practice^a^	70/174 (40.2)	58/210 (27.6)	12.6 (3.2 to 22.0)
Left to small practice^b^	104/174 (59.8)	152/210 (72.4)	−12.6 (−22.0 to −3.2)

Abbreviation: PE, private equity.

^a^ Defined as working elsewhere (ie, no longer observed at the original practice prior to PE exit) and observed at a practice with over 120 physicians.

^b^ Defined as working elsewhere and observed only at practices with 120 or fewer physicians.

## Discussion

PE firms have purchased large numbers of US physician practices. Given their objective of realizing a return by selling acquired practices within roughly 3 to 7 years, understanding how these exits impact physicians and physician market structure is highly relevant to physicians, regulators, investors, and patients. In this case-control study, we found that physicians employed in PE-owned practices in which the owners exited between 2016 and 2018 were 16.5 percentage points less likely to be working in the practice 2 years after exit than were a matched set of physicians in similarly sized practices, in the same specialty, and located in the same HRR but whose practice did not experience a PE exit during that period. Given the 60% mean rate of staying among controls, this represents a 27.5% reduction in retention.

Prior research has documented increased physician turnover following initial acquisition by a PE firm. Bruch et al^10^ found departure rates were 6 percentage points higher for physicians at previously independent practices acquired by PE firms between 2014 and 2019 than among matched controls. Our finding of a 16.5–percentage point increase in departures after exit, while not strictly comparable to Bruch et al^10^ because the control group in that study was limited expressly to physicians working in independent practices, suggests that the aggregate long-term effect of PE investment is to reduce physician retention.

The increase in turnover after PE exit occurred despite most exiting practices being purchased by PE investors, who may offer financial incentives to encourage retention. The finding of faster turnover at practices purchased by non-PE investors (ie, more turnover in the first year after exit) could be consistent with PE buyers providing such incentives; however, by 2 years after exit, the difference in turnover between practices purchased by PE firms and non-PE firms became insignificant. This may be of note to investors, who may expect greater retention.

There are several potential reasons for elevated turnover after PE exit. The data do not support the hypothesis that physicians departing after an initial PE exit received a payout that induced retirement. One alternative explanation for higher turnover is that physicians may have found it less rewarding to stay after PE exit, perhaps because the expected return from any equity stakes in later-stage PE-backed investments is typically lower. In addition, practices sold by PE tend to have fewer assets and more liabilities, reducing potential investment in practice-related activities and heightening the risk of subsequent instability (eg, bankruptcy). Relatedly, once the first PE-ownership group has realized the returns from picking low-hanging fruit, the next owners may lean on physicians to engage in margin-boosting activities that could undermine physicians’ autonomy and morale.^10,11^ Physicians may want to consider these possibilities before involving PE in their practice.

Turnover has implications for practices, patients, and physicians themselves. For practices, turnover can be disruptive financially and operationally.^18,20,21^ For patients who switch physicians due to turnover, this transition can impact experience, clinical outcomes, and utilization. Turnover has also been found to lead to increased use of higher-cost care.^16^ Physicians may benefit from changing employers, moving to a more preferred practice setting, or obtaining more favorable terms of employment^22^; however, the nudge from an ownership change implies a “push” rather than a “pull” motivation for changing and does not necessarily imply improved conditions. Last, of note for regulators, the finding that physicians in PE-exiting practices were 10.1 percentage points likelier than controls to leave for large physician practices suggests PE investment in physician markets facilitates consolidation of physician markets not only when practices are acquired and rolled up but also when they are sold. In light of research showing that physician consolidation tends to yield higher prices with no improvement in quality,^23,24^ this effect is important to incorporate when considering costs and benefits of PE involvement in physician markets.

### Limitations

This study had several limitations. First, our data were limited to physicians filing Medicare fee-for-service claims; physicians who billed Medicare in the baseline year but not in subsequent years will be incorrectly classified as retired. In eTable 4 in Supplement 1, we present evidence that the measurement error attributable to mislabeled retirement is similar in magnitude for the treatment and control groups, suggesting this limitation is unlikely to bias our results.

Second, despite efforts made to identify the universe of PE exits, some may have been excluded from our sample. Although PitchBook is the best available data source to identify PE exits, it is incomplete. Unintentional inclusion of PE-exiting practices and affiliated physicians in the control group should bias results toward the null.

Third, physicians in the data are sometimes affiliated with multiple practices, and a primary practice for each physician is not identified. Thus, physicians in the treatment group may be affiliated with other practices in addition to a PE-exiting practice both prior to and following exit; our focus is on whether they ceased to be affiliated with the PE-exiting practice after exit. We are unaware of reasons why such multihoming would affect the results.

Fourth, some turnover may be associated with ownership change in general rather than sale by a PE firm specifically. Some members of the control group may be in practices experiencing ownership changes. Our data do not allow us to limit the control group to physicians in practices without any ownership changes. Thus, the results are best interpreted as capturing the effects of PE exit on turnover compared with natural turnover among physicians in non–PE-exiting practices during the same time period, controlling for differences in specialty, geographic area, practice size, and graduation cohort.

Fifth, our strict matching criteria on specialty, HRR, and practice size limited the treatment group to 405 of 722 PE-exiting physicians. While these criteria reduce omitted variable bias, they could introduce selection bias. However, as shown in eFigure 2 in Supplement 1, results are similar when we include virtually all PE-exiting physicians (700 of 722) by matching on Census division instead of HRR. These findings dampen concerns about both potential sources of bias.

## Conclusions

Results of this case-control study complement prior research showing that PE acquisition leads to higher rates of physician departure. The finding that departure rates are also elevated after PE exits is an important consideration for multiple stakeholders. Because physicians who leave are likely to join larger practices, and those departing PE-exiting practices even more so, PE exits may heighten concentration of physician markets. These findings are also likely to be relevant to potential investors, as turnover can impact organizational performance. Importantly, patients who change physicians due to turnover may also be negatively impacted. Additional study of PE exits, including potential impacts on patient care and impact of final exits to non-PE buyers, would help solidify our understanding of the long-term effects of PE on health care stakeholders.

## Notes

Supplement 1. **eMethods.** Data Analyses **eTable 1.** Characteristics of PE-Exited Practices and Practice Sites **eTable 2.** Employment Decisions of Physicians in Private Equity (PE)–Exiting Practices Relative to Controls, Before and After PE Exit Tabular Results **eTable 3. **Physician Employment Decisions Among Physicians Leaving to Work Elsewhere After PE Exit **eTable 4.** Physician Reappearance to Sample After Classified as Retired **eFigure 1.** Employment Decisions of Physicians in Private Equity (PE)–Exiting Practices Relative to Controls, Before and After PE Exit by Graduation Year Before and After 1990 **eFigure 2.** Employment Decisions of Physicians in Private Equity (PE)–Exiting Practices Relative to Controls, Before and After PE Exit: Robustness to Alternative Number of Matched Controls **eFigure 3.** Employment Decisions of Physicians in Private Equity (PE)–Exiting Practices Relative to Controls, Before and After PE Exit: Robustness to Alternative Matching Geography **eFigure 4.** Employment Decisions of Physicians in Private Equity (PE)–Exiting Practices Relative to Controls, Before and After PE Exit: Robustness to Clustering at Practice Level **eFigure 5.** Employment Decisions of Physicians in Private Equity (PE)–Exiting Practices Relative to Controls, Before and After PE Exit: Robustness to Limiting the Control Group to Physicians in Practices With 50 or Fewer Physicians **eFigure 6.** Employment Decisions of Physicians in Private Equity (PE)–Exiting Practices Relative to Controls, Before and After PE Exit: Robustness to Limiting the Control Group to Physicians in Practices With Below-Median Size Within Specialty **eFigure 7.** Employment Decisions of Physicians in Private Equity (PE)–Exiting Practices Relative to Controls, Before and After PE Exit by Physician Specialty

Supplement 2.
Data Sharing Statement
