# Supplementary material for: Sale of Private Equity–Owned Physician Practices and Physician Turnover
Source: JAMA Health Forum. 2025 Feb 14;6(2):e245376. doi: 10.1001/jamahealthforum.2024.5376 (PMC11829224; doi:10.1001/jamahealthforum.2024.5376)
Supplement: Supplement 1. — eMethods. Data Analyses eTable 1. Characteristics of PE-Exited Practices and Practice Sites eTable 2. Employment Decisions of Physicians in Private Equity (PE)–Exiting Practices Relative to Controls, Before and After PE Exit Tabular Results eTable 3. Physician Employment Decisions Among Physicians Leaving to Work Elsewhere After PE Exit eTable 4. Physician Reappearance to Sample After Classified as Retired eFigure 1. Employment Decisions of Physicians in Private Equity (PE)–Exiting Practices Relative to Controls, Before and After PE Exit by Graduation Year Before and After 1990 eFigure 2. Employment Decisions of Physicians in Private Equity (PE)–Exiting Practices Relative to Controls, Before and After PE Exit: Robustness to Alternative Number of Matched Controls eFigure 3. Employment Decisions of Physicians in Private Equity (PE)–Exiting Practices Relative to Controls, Before and After PE Exit: Robustness to Alternative Matching Geography eFigure 4. Employment Decisions of Physicians in Private Equity (PE)–Exiting Practices Relative to Controls, Before and After PE Exit: Robustness to Clustering at Practice Level eFigure 5. Employment Decisions of Physicians in Private Equity (PE)–Exiting Practices Relative to Controls, Before and After PE Exit: Robustness to Limiting the Control Group to Physicians in Practices With 50 or Fewer Physicians eFigure 6. Employment Decisions of Physicians in Private Equity (PE)–Exiting Practices Relative to Controls, Before and After PE Exit: Robustness to Limiting the Control Group to Physicians in Practices With Below-Median Size Within Specialty eFigure 7. Employment Decisions of Physicians in Private Equity (PE)–Exiting Practices Relative to Controls, Before and After PE Exit by Physician Specialty [file jamahealthforum-e245376-s001.pdf]

# Supplementary Online Content

Berquist V, Klarnet L, Dafny L. Sale of private equity–owned physician practices and physician turnover. *JAMA Health Forum*. 2025;6(2):e245376. doi:10.1001/jamahealthforum.2024.5376

**eMethods.** Data Analyses

**eTable 1.** Characteristics of PE-Exited Practices and Practice Sites

**eTable 2.** Employment Decisions of Physicians in Private Equity (PE)–Exiting Practices Relative to Controls, Before and After PE Exit Tabular Results

**eTable 3.** Physician Employment Decisions Among Physicians Leaving to Work Elsewhere After PE Exit

**eTable 4.** Physician Reappearance to Sample After Classified as Retired

**eFigure 1.** Employment Decisions of Physicians in Private Equity (PE)–Exiting Practices Relative to Controls, Before and After PE Exit by Graduation Year Before and After 1990

**eFigure 2.** Employment Decisions of Physicians in Private Equity (PE)–Exiting Practices Relative to Controls, Before and After PE Exit: Robustness to Alternative Number of Matched Controls

**eFigure 3.** Employment Decisions of Physicians in Private Equity (PE)–Exiting Practices Relative to Controls, Before and After PE Exit: Robustness to Alternative Matching Geography

**eFigure 4.** Employment Decisions of Physicians in Private Equity (PE)–Exiting Practices Relative to Controls, Before and After PE Exit: Robustness to Clustering at Practice Level

**eFigure 5.** Employment Decisions of Physicians in Private Equity (PE)–Exiting Practices Relative to Controls, Before and After PE Exit: Robustness to Limiting the Control Group to Physicians in Practices With 50 or Fewer Physicians

**eFigure 6.** Employment Decisions of Physicians in Private Equity (PE)–Exiting Practices Relative to Controls, Before and After PE Exit: Robustness to Limiting the Control Group to Physicians in Practices With Below-Median Size Within Specialty

**eFigure 7.** Employment Decisions of Physicians in Private Equity (PE)–Exiting Practices Relative to Controls, Before and After PE Exit by Physician Specialty

This supplementary material has been provided by the authors to give readers additional information about their work.

© 2025 Berquist V et al. *JAMA Health Forum*.

**eMethods.** Data Analyses

**Data Processing Methodology**

This section describes the construction of our dataset. PE exits were identified using a proprietary database from PitchBook Inc, a market intelligence firm that tracks mergers and acquisitions across all industries including healthcare. Exits are considered by PitchBook to be any sale that results in a change of majority ownership (or a public listing). In practice, none of the exits in our data were public listings. All PE exits (i.e., “sales” in PitchBook) between the years 2016-2018 and tagged with the primary industry of ‘clinics/outpatient services’ in the US were examined. Practices were excluded if they were in industries not primarily managed by physicians – for example, dentistry, addiction/rehabilitation, physical therapy, and non-psychiatric behavioral health. Exits were manually examined to determine if they were the first exit by a PE firm – that is, sales were only included if the practices had been previously acquired by a PE firm *only once*, and if their first sale by that PE firm occurred during the period 1/2016 to 12/2018, inclusive. Exits were excluded if they were not the exit by the first PE owner. To ensure accuracy of the data, the company and year of exit were manually confirmed using publicly available online materials and corrections were made when necessary. To the extent that exits occurred that were not recorded in the PitchBook data, these omissions can be expected to attenuate the results as some control physicians would in fact belong in the treatment group.

Practices were identified in the CMS National Downloadable File (NDF) using the group practice ID assigned by PECOS (Provider Enrollment, Chain, and Ownership System). The NDF linked a physician to a practice if the physician billed at least one Medicare fee-for-service claim through the practice within the prior six months. Multisite practices recorded under the same group practice ID are considered one practice. Physicians may bill from more than one practice. Practices under common ownership (i.e., owned by the same company) may have separate group practice IDs if they operate independently, most likely due to operating in different geographic areas or under distinct administrative processes with decentralized systems for billing, accounting, and financial reporting. Whether a company operates under a single or multiple group practice IDs is ultimately determined by how they enrolled in PECOS, which may also reflect strategic considerations.

Companies that experienced PE exit were linked to practices in the NDF using several methods. Matches were primarily found by matching practice and organization names in the NDF to the trade names of the companies. Where this was not possible, generally due to names in the NDF database being different from trade names, matching was conducted manually using exact addresses and physician names.

An example of this methodology is as follows. PitchBook documented the sale by PE firm Sagard Holdings of Vein Clinics of America in December 2017. Vein Clinics of America was identified in the NDF under its organization’s name and owned ten separate practices operating in different states. This transaction constituted one exit and accounted for 10 PE-exiting practices.

To form a control group, we similarly assigned eligible controls to their associated practices in each year prior to the exit years (2015, 2016, and 2017). To calculate practice size, we used the sum of unique NPIs of physicians billing through the practice. We then matched physician-

© 2025 Berquist V et al. *JAMA Health Forum*.

practice pairs in the treatment group to physician-practice pairs in the control group. Once a physician-practice is matched to a treatment physician, the matched physician was precluded from matching to any other treatment physician. This procedure yielded a dataset of PE-exited physicians and matched controls unique at the physician level, where each physician is assigned a single "focal" practice.

A physician was coded as staying in the focal practice if they continued to bill at a practice that matched any of the following attributes of the focal practice: group practice ID, organization name, or street address. This approach addresses a potential concern with relying solely on group practice IDs, as practices may change their group practice IDs to match an acquirer's ID after an ownership transition, which could misclassify PE-exited physicians as having left the practice. This approach leverages practice-level information, capturing if a physician moves location but stays within the practice, as well as location information, capturing if a practice changes organization name and group practice ID but the physician continues to bill from the same location. All practices for a given physician-year were considered for this classification. If a physician began billing at a new practice, but continued to bill from the focal practice, they would be coded as "stayed." The key assumption is that the new ownership does not change the practice ID, organization name, *and* address of the physician's initial practice site.

To validate this methodology, we manually reviewed all employment classifications for a large sample of treatment physicians (specifically, all physicians affiliated with PE-owned practices as of 2015; this was the treatment sample when we initiated this study). For this sample - which comprises 70% of the treatment sample we ultimately used – we used online sources to document the ownership status of the practices they billed from in 2019. Out of the 1,401 physician destinations reviewed, only 1% appeared to be misclassified. Although we later modified the sample (specifically, to include physicians affiliated with PE-exiting practices in the year prior to PE exit, as opposed to only physicians affiliated in 2015), this thorough initial review provides confidence that the risk of misclassified physician employment destinations is low.

Additional data on physician and practice characteristics were identified in the NDF. These include physician graduation year, physician specialty, size of practice, practice HRR, and practice census division. eTable 1 presents descriptive statistics of the counts of practices and practice-sites by year of PE exit, year of PE purchase, and specialty.

**Robustness Checks**

We conducted four robustness checks using different matching requirements and sample selection criteria. The first robustness check expanded the number of required matched controls for each PE-exited physician from two to five. This restriction reduced the sample of PE-exited physicians from 405 to 296 but increased the size of the control group from 810 to 1,480.

The second robustness check relaxed the geographic match requirement of an exact match on HRR to an exact match on census division. Compared to HRR, census division is a much larger geographic area; the US has 306 HRRs and only 9 census divisions. By loosening the geographic match requirement, we obtained two matched controls for 700 PE-exited physicians in the treatment group, nearly all of the 722 PE-exited physicians we observe.

© 2025 Berquist V et al. *JAMA Health Forum*.

The third and fourth robustness checks tested the sensitivity of the results to excluding large practices from the control group. The objective of these sample restrictions is to limit the frequency of academic medical systems or corporate employers in the control group, as turnover trends for these employees could differ from turnover trends among physicians in independent or PE-backed practices. The third check restricted physicians in the control group to practices with at most 50 physicians, as opposed to the 120-physician maximum size restriction in the primary sample. The fourth check limited the control group to physicians at practices with a below-median number of physicians by specialty. For example, the median practice size for dermatology practices was 6 physicians; the fourth robustness check limits the control group for dermatology practices to those with fewer than 6 physicians. This check flexibly removes large practices by allowing the maximum threshold to vary by specialty.

eSupplement eFigures 2, 3, 4, and 5 present the results of these four robustness checks of the multinomial logit regression reported in Figure 1. All estimates were quantitatively similar to the results from the primary sample. For example, in the primary sample PE-exited physicians were 16.5 percentage points more likely (95% CI, 10.9 to 22.1) to work elsewhere two years after exit than controls. In the first robustness check, this number was 13.8 percentage points (95% CI, 7.9 to 19.6), and in the second, it was 18.0 percentage points (95% CI, 13.8 to 22.1). In the third check this number was 13.6 percentage points (95% CI, 7.7 to 19.5), while in the fourth, it was 14.5 percentage points (95% CI, 7.7 to 21.3). These robustness checks show the results are robust to specifications increasing the stringency of matching, as well as a specification reducing the stringency of matching.

**Heterogeneity Analyses**

We also tested for heterogeneity in treatment effects based on whether the buyer was a PE firm (Figure 2), the physician's age (eFigure 1), and the physician's specialty (eFigure 7). The methodology for analyzing the effects by buyer is outlined in detail below. Modifications for the other heterogeneity analyses are also described.

Our objective was to obtain separate estimates of the employment decisions for physicians at practices acquired by PE buyers and those acquired by non-PE buyers, as compared to control group physicians. This was done using a single, pooled multinomial logit regression where the indicators for PE exit in each year relative to exit were interacted with an indicator for PE buyer (panel (a)) and an indicator for non-PE buyer (panel (b)). This regression included controls for graduation decade and year relative to exit.

The estimating equation can be written:

$$Y_{it} = \sum_{k\in\{-2,0,1,2\}} \beta_k^{PE} D_{i,t-k} 1\{PE\ Buyer_i = 1\} + \sum_{k\in\{-2,0,1,2\}} \beta_k^{non-PE} D_{i,t-k} 1\{PE\ Buyer_i = 0\} + \sum_{k\in\{-2,0,1,2\}} \gamma_k 1\{t - exit\ year_i = k\} + \alpha X_i + \varepsilon_{it}$$

© 2025 Berquist V et al. *JAMA Health Forum*.

where k is the year relative to exit (i.e., event time), t is the calendar year, and i indicates a physician. $D_{i,t}$ is an indicator variable that equals one if a physician experiences a PE exit in year t. $Y_{it}$ is a categorical outcome that indicates whether the physician stayed, worked elsewhere, or retired. $X_i$ is a vector of graduation decade fixed effects (<1980, 1981-1990, 1991-2000, 2001+). The coefficients of interests are $\beta_k^{PE}$ (which capture the leads and lags for PE exit to PE buyers) and $\beta_k^{non-PE}$ (which capture the leads and lags for PE exit to non-PE buyers).

To test whether the differences between the coefficients for PE buyers and non-PE buyers were statistically significant, we estimated another single, pooled regression with indicators for PE exit in each year relative to exit, as well as interaction terms between a non-PE buyer indicator and PE exit for each year relative to exit. In panel (c), we plotted the coefficient for this interaction term, which directly tests the difference between the coefficients for non-PE buyers and PE buyers.

This estimating equation can be written:

$$Y_{it} = \sum_{k \in \{-2,0,1,2\}} \beta_k^{PE} D_{i,t-k} + \sum_{k \in \{-2,0,1,2\}} \beta_k^{interact} D_{i,t-k} 1\{PE\ Buyer_i = 0\} + \sum_{k \in \{-2,0,1,2\}} \gamma_k 1\{t - exit\ year_i = k\} + \alpha X_i + \varepsilon_{it}$$

where k is the year relative to exit (i.e., event time), t is the calendar year, and i indicates a physician. $D_{i,t}$ is an indicator variable that equals one if a physician experiences a PE exit in year t. $Y_{it}$ is a categorical outcome that indicates whether the physician stayed, worked elsewhere, or retired. $X_i$ is a vector of graduation decade fixed effects (<1980, 1981-1990, 1991-2000, 2001+). The coefficients of interest are $\beta_k^{interact}$, which reflect the difference in outcomes for physicians exiting to PE and non-PE buyers, relative to physicians not in exiting practices.

To examine heterogeneity by physician graduation year or by specialty, we replaced the indicator functions $1\{PE\ Buyer_i = 1\}$ and $1\{PE\ Buyer_i = 0\}$ with corresponding indicator functions for each graduation year bin or specialty. For these heterogeneity analyses, we also included interactions between the year-relative-to-exit controls and the indicators for graduation year bin or specialty. These additional interactions account for any time-varying trends that are age- or specialty- specific. For instance, as years increase relative to treatment, older physicians' probability of retirement should increase faster relative to younger physicians, as the former group approaches retirement age. Without these age-specific time controls, we might incorrectly attribute a spurious effect on retirement for older physicians by comparing them to the average across all control physicians.

We did not include separate year-relative-to-exit controls interacted with PE and non-PE buyer indicators. This decision was made because we do not have information on which control physicians would have been counterfactually acquired by PE or non-PE buyers. As a result, applying such interactions would not be meaningful in this context.

eFigure 7 presents the heterogeneity results broken out by the two most common specialties in our primary sample of PE-exiting physicians – family medicine and dermatology – each

© 2025 Berquist V et al. *JAMA Health Forum*.

comprising 94 of the 405 treatment physicians. Two years after PE exit, family medicine physicians were 14.7 percentage points less likely to stay at the PE-exiting practice than physicians in the treatment group in all other specialties (excluding dermatology) (95% CI, 0.02 to 29.3). For dermatologists, this difference was 14.1 percentage points, and statistically significant at p=0.051. (95% CI, -0.1 to 28.3). eFigure 1 shows no statistically significant differences in the likelihood of staying, working elsewhere, or retiring between treatment physicians who graduated before 1990 and those who graduated after 1990.

**Analysis of Measurement Error in Retirement**

We classify a physician as retired if they appeared in the NDF in year *t* and do not appear in the NDF in year *t+1*, indicating that they did not bill Medicare in the last six months of the year *t+1*. Since retirement is not directly observed in the data, some physicians classified as retired may have simply stopped billing Medicare rather than actually retiring. To assess the extent of this potential measurement error, and to understand how it varies between treatment and control physicians, we analyze the proportion of physicians classified as "retired" who subsequently reappear in the data in later years.

eTable 4 presents the share of physician-years classified as retired that reappear in the NDF in subsequent years, broken down by treatment and control groups. Since we require all physicians to have billed Medicare in the year prior to exit, we limit this analysis to the year of PE exit and later. We also exclude the year 2020 to ensure that we can observe whether physicians billed at least one year after the year we classify them as retired, i.e. physicians absent in the NDF in 2019 could be identified as present again in 2020, the last year for which we have the NDF.

Among the primary sample of treatment and control physicians, 8.1% of physician-years classified as retired ultimately reappear in the data in subsequent years. This rate is 6.2% for treatment physicians and 8.9% for control physicians; however, the difference between the treatment and control groups is not statistically significant (95% CI -8.1% to 2.7%). If treatment physicians were less likely than controls to be falsely classified as retired, that would bias the results away from finding a positive effect of PE exit on retirement. However, to bound the magnitude of this conservative bias, consider the implications if the true difference were at the lower bound of the confidence interval, i.e. 8.1%. This would imply approximately 4.5 fewer retirements two years after exit in the treatment group than observed (0.081 * 55). This would reduce the share retiring in the treatment group two years after exit from 13.6% to 12.5%, suggesting an upper bound on the bias from this source of measurement error is 1.1 percentage points.

Unfortunately, we are unable to assess the potential bias due to another potential source of measurement error for retirement – if a physician stopped billing Medicare but continued to care for other patient populations. However, the share of physicians formally opting out of Medicare in the U.S. is 1.1%, with only slightly higher rates among the two largest specialties in our sample (family medicine at 1.6% and dermatology at 1.2%).[1]

[1] https://www.kff.org/medicare/issue-brief/how-many-physicians-have-opted-out-of-the-medicare-program/#:~:text=Few%20(1.1%25)%20Physicians%20Have,Out%20of%20Medicare%20in%202023&text=While%20the%20overall%20opt%2Dout,and%20plastic%20and%20reconstructive%20surgery.

© 2025 Berquist V et al. *JAMA Health Forum*.

**eTable 1.** Characteristics of PE-Exited Practices and Practice Sites

| | Count of Practices | | Count of Practice Sites | |
|---|---|---|---|---|
| | All PE-Exited Practices | Primary Sample | All PE-Exited Practice Sites | Primary Sample |
| **Year of PE Exit** | | | | |
| 2016 | 29 | 26 | 56 | 44 |
| 2017 | 35 | 34 | 96 | 73 |
| 2018 | 10 | 10 | 35 | 35 |
| **Year of PE Purchase** | | | | |
| 2007 | 9 | 9 | 22 | 21 |
| 2008 | 2 | 2 | 2 | 2 |
| 2011 | 22 | 22 | 57 | 39 |
| 2012 | 4 | 4 | 9 | 9 |
| 2013 | 2 | 1 | 9 | 5 |
| 2014 | 3 | 3 | 21 | 12 |
| 2015 | 12 | 10 | 15 | 13 |
| 2016 | 15 | 14 | 39 | 38 |
| 2018 | 1 | 1 | 1 | 1 |
| Missing | 4 | 4 | 12 | 12 |
| **Specialty** | | | | |
| Dermatology | 22 | 19 | 45 | 33 |
| Family Medicine | 14 | 13 | 27 | 27 |
| Ophthalmology | 6 | 5 | 20 | 16 |
| Anesthesiology | 1 | 1 | 4 | 4 |
| Internal Medicine | 2 | 2 | 13 | 13 |
| Emergency Medicine | 4 | 5 | 6 | 6 |
| General surgery | 1 | 1 | 3 | 3 |
| Radiology | 2 | 3 | 8 | 7 |
| Pain management | 6 | 5 | 30 | 15 |
| Urology | 1 | 1 | 4 | 4 |
| Nephrology | 1 | 1 | 1 | 1 |
| Other | 14 | 14 | 26 | 23 |
| | | | | |
| Total | 74 | 70 | 187 | 152 |

Notes: This table compares characteristics of practices and practice sites among all 74 practices identified in the NDF and the 70 practices in our analytical sample. Practices are defined using the group practice ID from PECOS and practice sites are defined as unique addresses within a group practice ID. Year of PE purchase comes from PitchBook and is not available for four practices. Specialties are defined as the mode of physician specialties within the practice or practice site.

© 2025 Berquist V et al. *JAMA Health Forum*.

**eTable 2.** Employment Decisions of Physicians in Private Equity (PE)–Exiting Practices Relative to Controls, Before and After PE Exit Tabular Results

| | Stay | Work Elsewhere | Retire |
|---|---|---|---|
| Treatment (PE-exited) interacted with year relative to exit | | | |
| Treatment X two years prior to exit | 0.027 | 0.009 | -0.036 |
| | (-0.017 to 0.071) | (-0.026 to 0.045) | (-0.066 to -0.006) |
| Treatment X year of exit | 0.000 | 0.031 | -0.032 |
| | (-0.047 to 0.048) | (-0.009 to 0.072) | (-0.060 to -0.003) |
| Treatment X one year after exit | -0.083 | 0.091 | -0.008 |
| | (-0.140 to -0.026) | (0.040 to 0.142) | (-0.045 to 0.029) |
| Treatment X two years after exit | -0.165 | 0.165 | -0.000 |
| | (-0.223 to -0.106) | (0.109 to 0.221) | (-0.041 to 0.040) |
| Year relative to exit (reference - two years prior to exit) | | | |
| Year of exit | -0.020 | 0.032 | -0.013 |
| | (-0.288 to -0.197) | (0.158 to 0.258) | (0.004 to 0.065) |
| One year after exit | -0.146 | 0.136 | 0.010 |
| | (-0.190 to -0.103) | (0.090 to 0.183) | (-0.017 to 0.037) |
| Two years after exit | -0.242 | 0.208 | 0.034 |
| | (-0.065 to 0.026) | (-0.015 to 0.080) | (-0.040 to 0.014) |
| Year of graduation (reference - ≤ 1980) | | | |
| 1981-1990 | 0.020 | 0.076 | -0.096 |
| | (-0.037 to 0.078) | (0.031 to 0.121) | (-0.139 to -0.053) |
| 1991-2000 | 0.034 | 0.058 | -0.091 |
| | (-0.022 to 0.089) | (0.015 to 0.101) | (-0.133 to -0.050) |
| ≥ 2001 | -0.069 | 0.124 | -0.056 |
| | (-0.123 to -0.014) | (0.083 to 0.166) | (-0.098 to -0.014) |

Notes: This table presents the marginal effects of PE exit on the probability of physician employment decisions from multinomial logit regressions underlying Figure 1. 95% confidence intervals are in parentheses.

© 2025 Berquist V et al. *JAMA Health Forum*.

**eTable 3.** Physician Employment Decisions Among Physicians Leaving to Work Elsewhere After PE Exit

| Employment Outcome Conditional on Working Elsewhere | PE-Exited Physicians | Matched Physicians | Difference | 95% CI on Difference |
|---|---|---|---|---|
| Two Years Before Exit | | | | |
| Left to Large Practice | 6 (14.3%) | 13 (17.3%) | -3.0% | (-17.1% to 11.0%) |
| Left to Small Practice | 36 (85.7%) | 62 (82.7%) | 3.0% | (-11.0% to 17.1%) |

Notes: This table presents unadjusted physician employment outcomes two years before PE exit for treatment relative to control physicians in the primary sample conditional on working elsewhere. Left to large practice is defined as working elsewhere (i.e., no longer observed at the original practice prior to PE exit) and observed at a practice with at least 120 physicians. Left to small practice is defined as working elsewhere and observed only at practices with fewer than 120 physicians.

© 2025 Berquist V et al. *JAMA Health Forum*.

**eTable 4.** Physician Reappearance to Sample after Classified as Retired

| Physician-Year Observations Classified as Retired that… | PE-Exited Physicians | Matched Physicians | Difference | 95% CI on Difference |
|---|---|---|---|---|
| Never Reappear in the NDF | 136 (93.8%) | 286 (91.1%) | 2.7% | (-2.7% to 8.1%) |
| Reappear in the NDF | 9 (6.2%) | 28 (8.9%) | -2.7% | (-8.1% to 2.7%) |

Notes: This table presents the share of physician-years that were classified as retired that ultimately reappear in the NDF in later years. The data is at the physician-year level and consists of the 405 treatment physicians and 810 control physicians in the primary sample. The data is further limited to physician-years classified as retired, which is defined as not observed in the NDF. Since we require all physicians to bill in the year prior to exit, we limit the sample to physician-years corresponding to the year of PE exit and later. We also exclude the 2020 year to be able to observe whether physicians billed a least one year after the year of retirement.

© 2025 Berquist V et al. *JAMA Health Forum*.

**eFigure 1.** Employment Decisions of Physicians in Private Equity (PE)–Exiting Practices Relative to Controls, Before and After PE Exit by Graduation Year Before and After 1990

**Panel a) Physician graduation year on or before 1990**

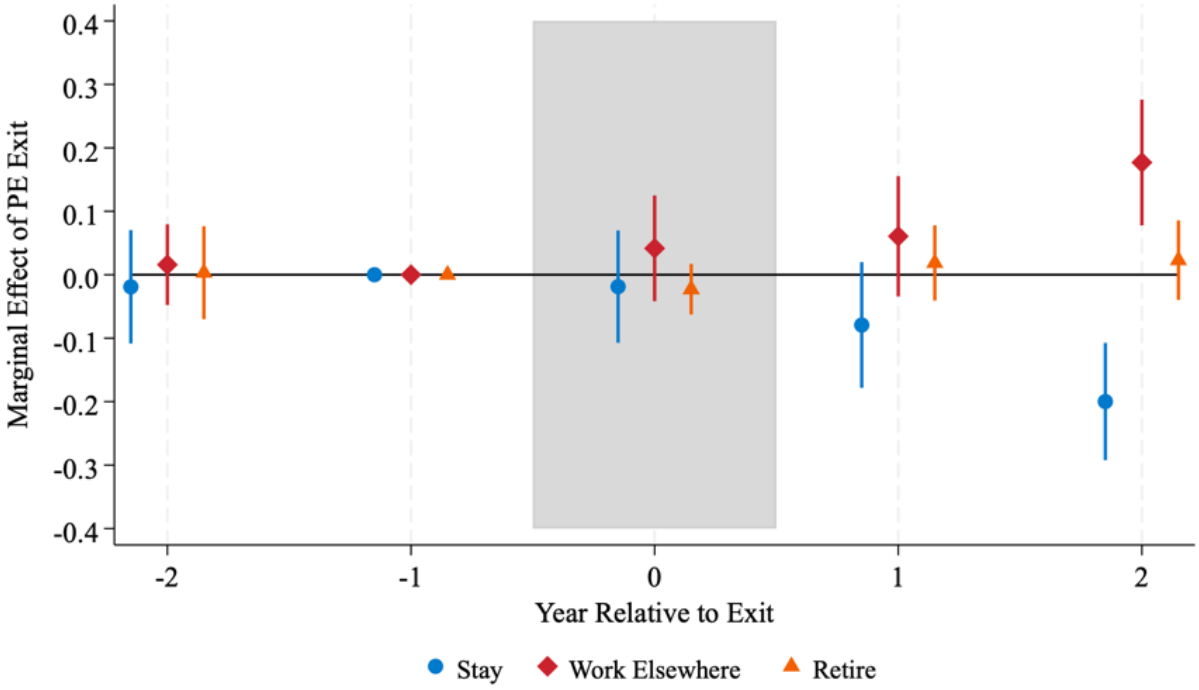


**Panel b) Physician graduation year after 1990**

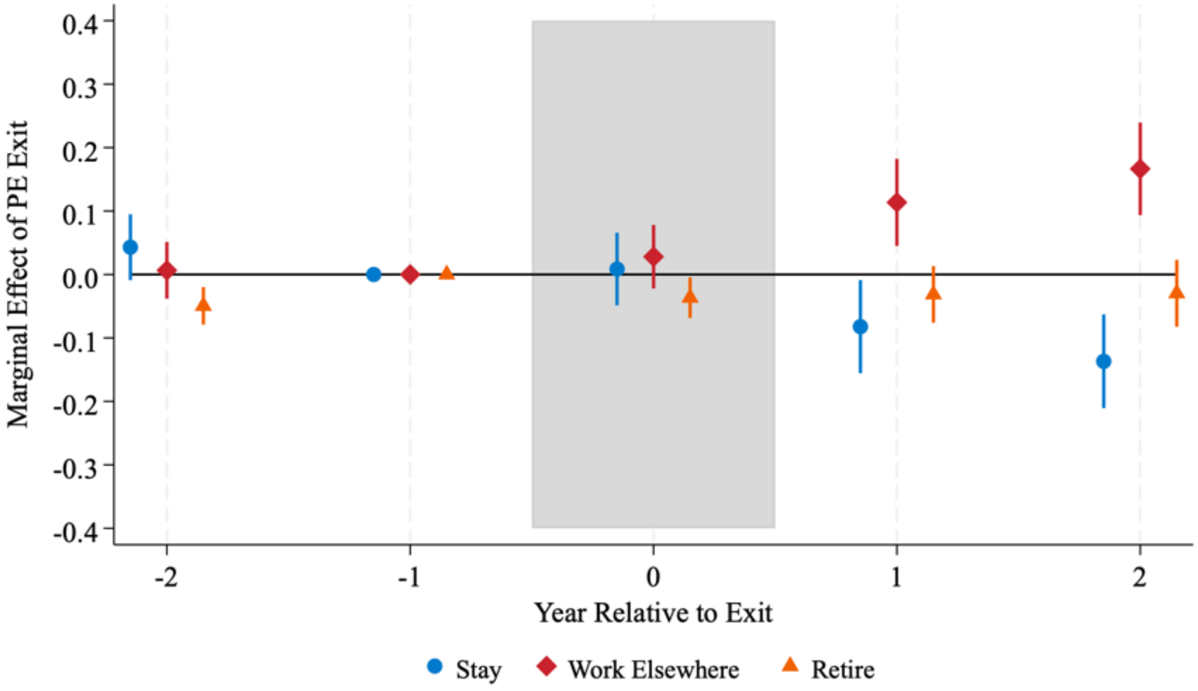


© 2025 Berquist V et al. *JAMA Health Forum*.

**Panel c) Difference: graduation after 1990 – graduation on or before 1990**

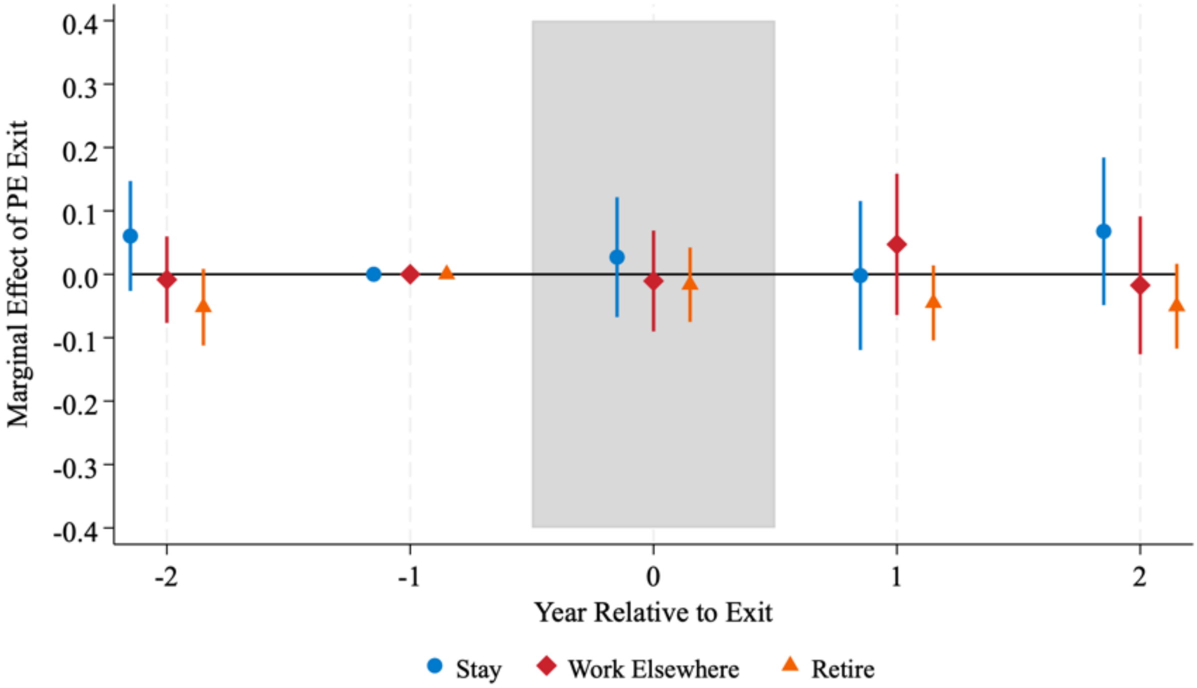


Notes: The figure plots the marginal effects from multinomial logit specifications for physician employment decisions in which the indicators for years relative to PE exit are interacted with indicators reflecting the timing of physician graduation. The data is at the physician-year level and point estimates for stay and retire are offset on the x-axis slightly for readability. The points represent the corresponding coefficients, and the vertical bars are 95% confidence intervals. Panels (a) and (b) plot the effects separately by whether the physician graduated before or after 1990; panel (c) plots the difference between these groups to illustrate whether they are statistically significant. Panels (a) and (b) are estimated using a single, pooled multinomial logit regression where a PE exit indicator for each year relative to exit is interacted separately with an indicator which equals one if the physician graduated on or before 1990 (coefficient estimates reported in panel (a)) and an indicator which equals one if the physician graduated after 1990 (coefficient estimates reported in panel (b)). Panel (c) plots the interaction term of a single, pooled multinomial logit regression with an indicator for PE exit in each year relative to exit and indicator for PE exit in each year relative to exit interacted with an indicator for graduation year after 1990. Both regressions control for the year relative to exit interacted with indicators for whether the physician graduated before or after 1990. The shaded region indicates the year of PE exit. Standard errors are clustered at the physician level.

© 2025 Berquist V et al. *JAMA Health Forum*.

**eFigure 2.** Employment Decisions of Physicians in Private Equity (PE)–Exiting Practices Relative to Controls, Before and After PE Exit: Robustness to Alternative Number of Matched Controls

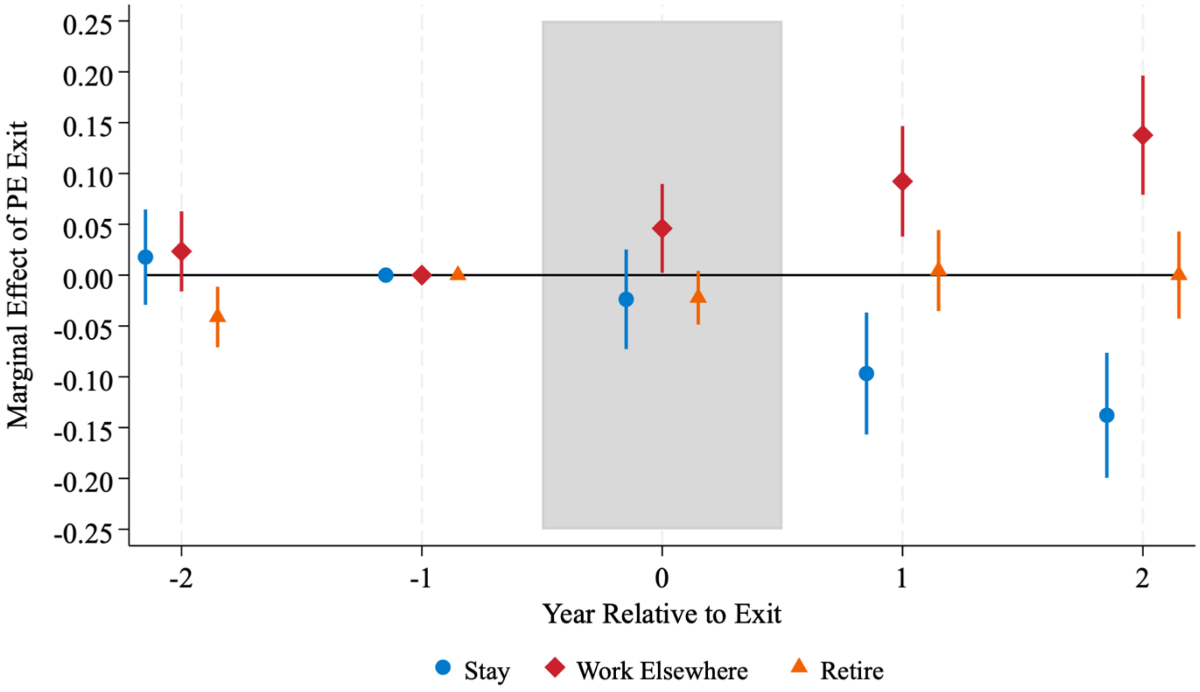


Notes: This figure is analogous to Figure 1, except requiring 5 controls for each PE-exited physician as opposed to 2. This specification includes 296 PE-exited physicians and 1,480 matched controls, compared to the primary sample which has 405 and 810, respectively. The figure plots the marginal effects from the multinomial logit specification for physician employment decisions. All coefficients are estimated in a single, pooled regression controlling for graduation decade and year relative to exit. The point estimates represent the corresponding coefficients, and the vertical bars are 95% confidence intervals. The data are at the physician-year level and point estimates for stay and retire are offset on the x-axis slightly for readability. The shaded region indicates the year of PE exit. Standard errors are clustered at the physician level.

© 2025 Berquist V et al. *JAMA Health Forum*.

**eFigure 3.** Employment Decisions of Physicians in Private Equity (PE)–Exiting Practices Relative to Controls, Before and After PE Exit: Robustness to Alternative Matching Geography

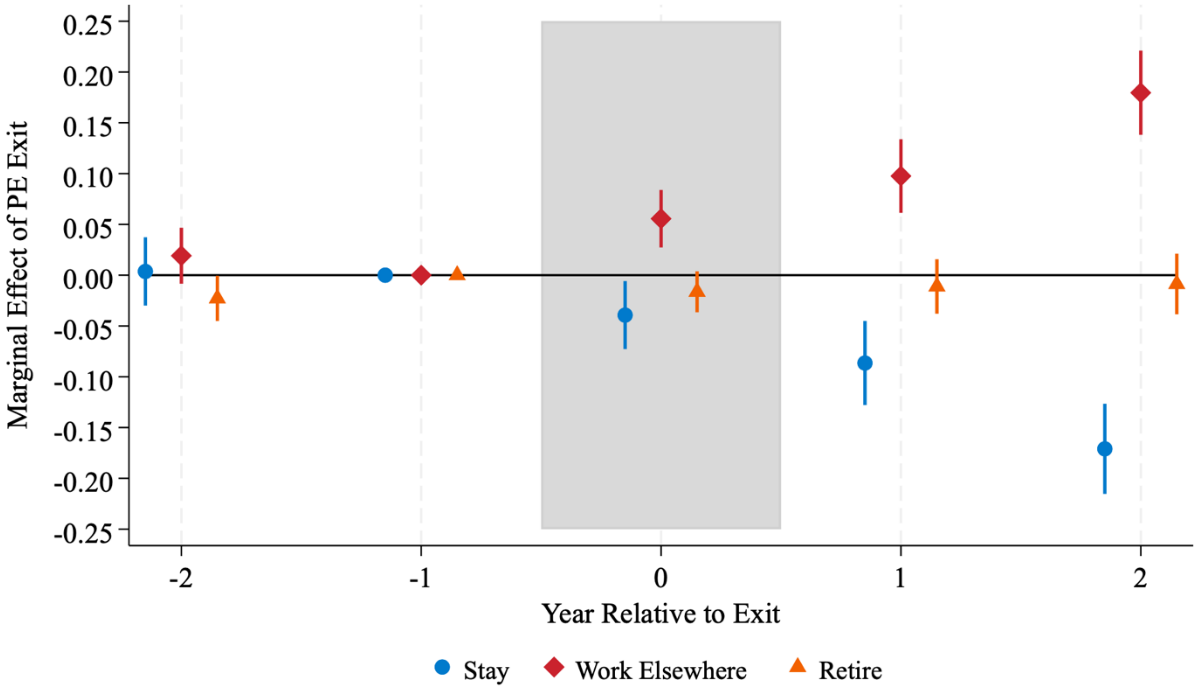


Notes: This figure is analogous to Figure 1, except requiring exact matching on census division, as opposed to HRR. This specification includes 700 PE-exited physicians and 1,400 matched controls, compared to the primary sample which has 405 and 810, respectively. The figure plots the marginal effects from the multinomial logit specification for physician employment decisions. All coefficients are estimated in a single, pooled regression controlling for graduation decade and year relative to exit. The point estimates represent the corresponding coefficients, and the vertical bars are 95% confidence intervals. The data are at the physician-year level and point estimates for stay and retire are offset on the x-axis slightly for readability. The shaded region indicates the year of PE exit. Standard errors are clustered at the physician level.

© 2025 Berquist V et al. *JAMA Health Forum*.

**eFigure 4.** Employment Decisions of Physicians in Private Equity (PE)–Exiting Practices Relative to Controls, Before and After PE Exit: Robustness to Clustering at Practice Level

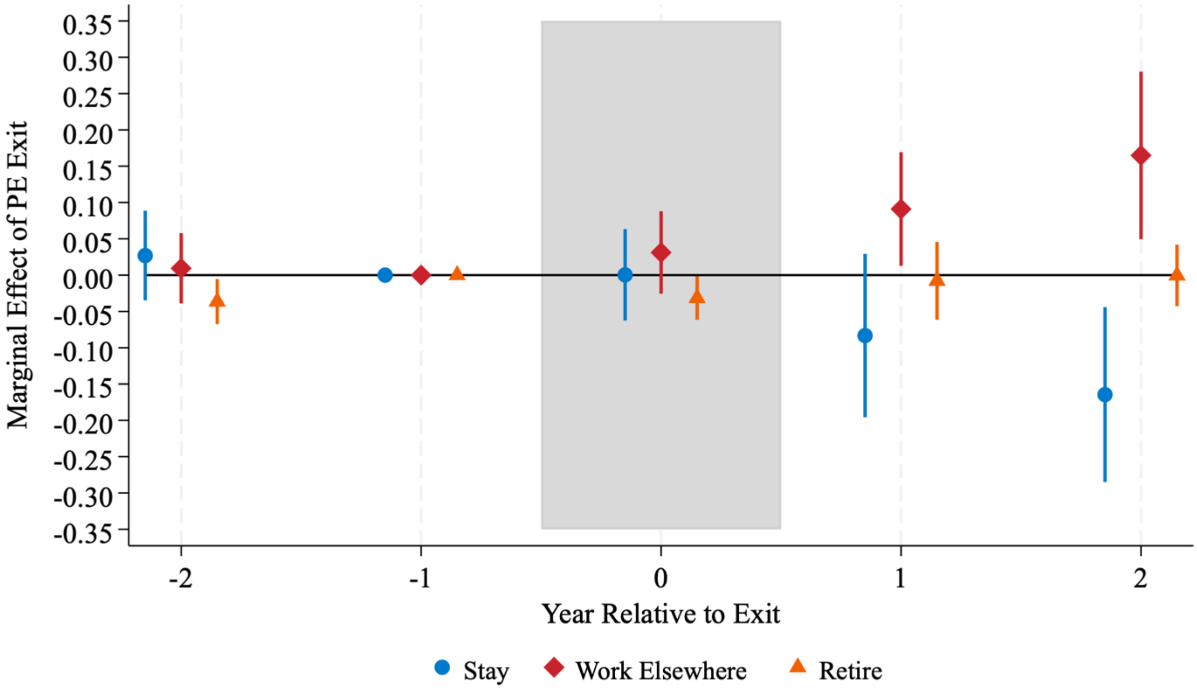


Notes: This figure is analogous to Figure 1, except clusters standard errors at the practice rather than provider level. The figure plots the marginal effects from the multinomial logit specification for physician employment decisions for the primary sample of 405 treatment physicians and 810 control physicians. All coefficients are estimated in a single, pooled regression controlling for graduation decade and year relative to exit. The point estimates represent the corresponding coefficients, and the vertical bars are 95% confidence intervals. The data are at the physician-year level and point estimates for stay and retire are offset on the x-axis slightly for readability. The shaded region indicates the year of PE exit.

© 2025 Berquist V et al. *JAMA Health Forum*.

**eFigure 5.** Employment Decisions of Physicians in Private Equity (PE)–Exiting Practices Relative to Controls, Before and After PE Exit: Robustness to Limiting the Control Group to Physicians in Practices With 50 or Fewer Physicians

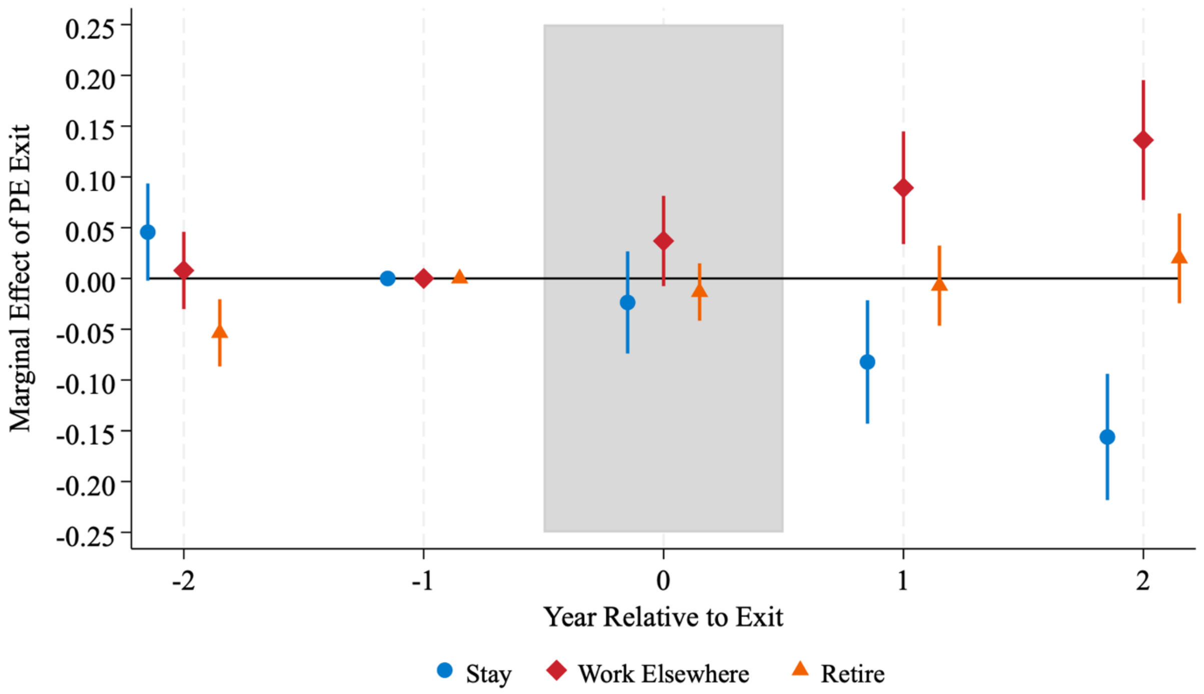


Notes: This figure is analogous to Figure 1, except the results are obtained from a sample that eliminates practices with 50 or more physicians from set of potential controls, as compared to the 120-physician restriction in the primary sample. This specification includes 359 PE-exited physicians, as compared to the primary sample which has 405. The figure plots the marginal effects from the multinomial logit specification for physician employment decisions. All coefficients are estimated in a single, pooled regression controlling for graduation decade and year relative to exit. The point estimates represent the corresponding coefficients, and the vertical bars are 95% confidence intervals. The data are at the physician-year level and point estimates for stay and retire are offset on the x-axis slightly for readability. The shaded region indicates the year of PE exit. Standard errors are clustered at the physician level.

© 2025 Berquist V et al. *JAMA Health Forum*.

**eFigure 6.** Employment Decisions of Physicians in Private Equity (PE)-exiting Practices Relative to Controls, Before and After PE Exit: Robustness to Limiting the Control Group to Physicians in Practices With Below-Median Size Within Specialty

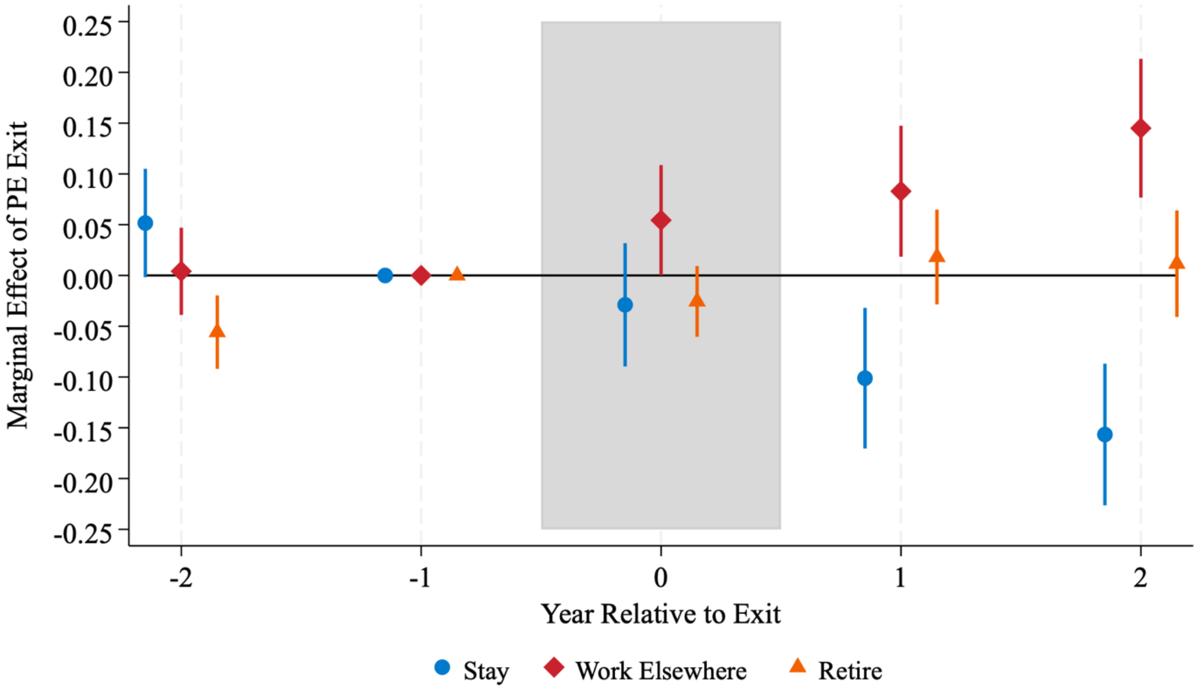


Notes: This figure is analogous to Figure 1, except the results are obtained from a sample that limits control physicians to those at practices with a below-median number of physicians by specialty. This specification includes 280 PE-exited physicians, as compared to the primary sample which has 405. The figure plots the marginal effects from the multinomial logit specification for physician employment decisions. All coefficients are estimated in a single, pooled regression controlling for graduation decade and year relative to exit. The point estimates represent the corresponding coefficients, and the vertical bars are 95% confidence intervals. The data are at the physician-year level and point estimates for stay and retire are offset on the x-axis slightly for readability. The shaded region indicates the year of PE exit. Standard errors are clustered at the physician level.

© 2025 Berquist V et al. *JAMA Health Forum*.

**eFigure 7.** Employment Decisions of Physicians in Private Equity (PE)–Exiting Practices Relative to Controls, Before and After PE Exit by Physician Specialty

**Panel a) Family medicine**

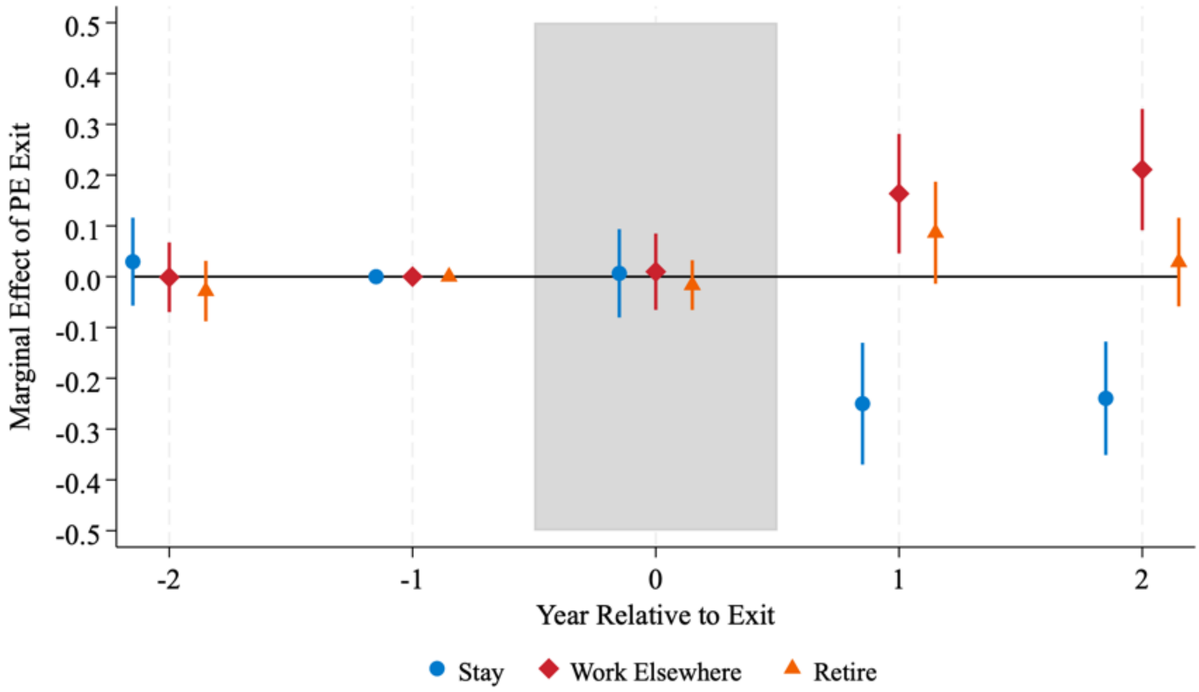


**Panel b) Dermatology**

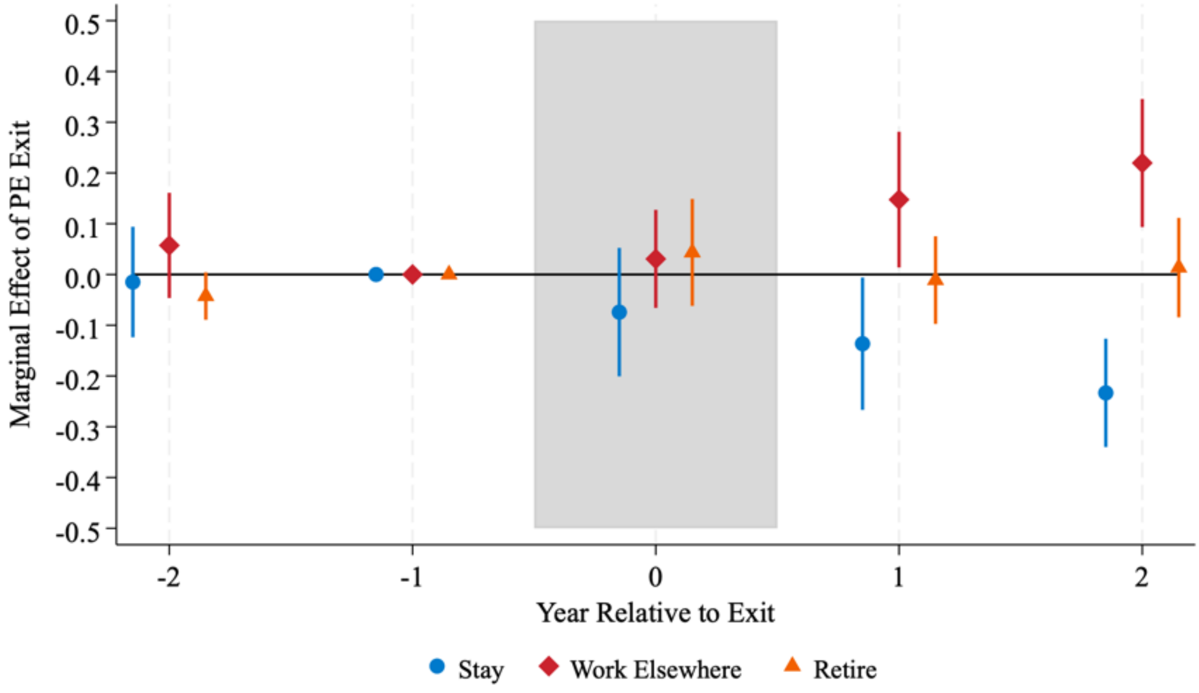


© 2025 Berquist V et al. *JAMA Health Forum*.

**Panel c) All other specialties**

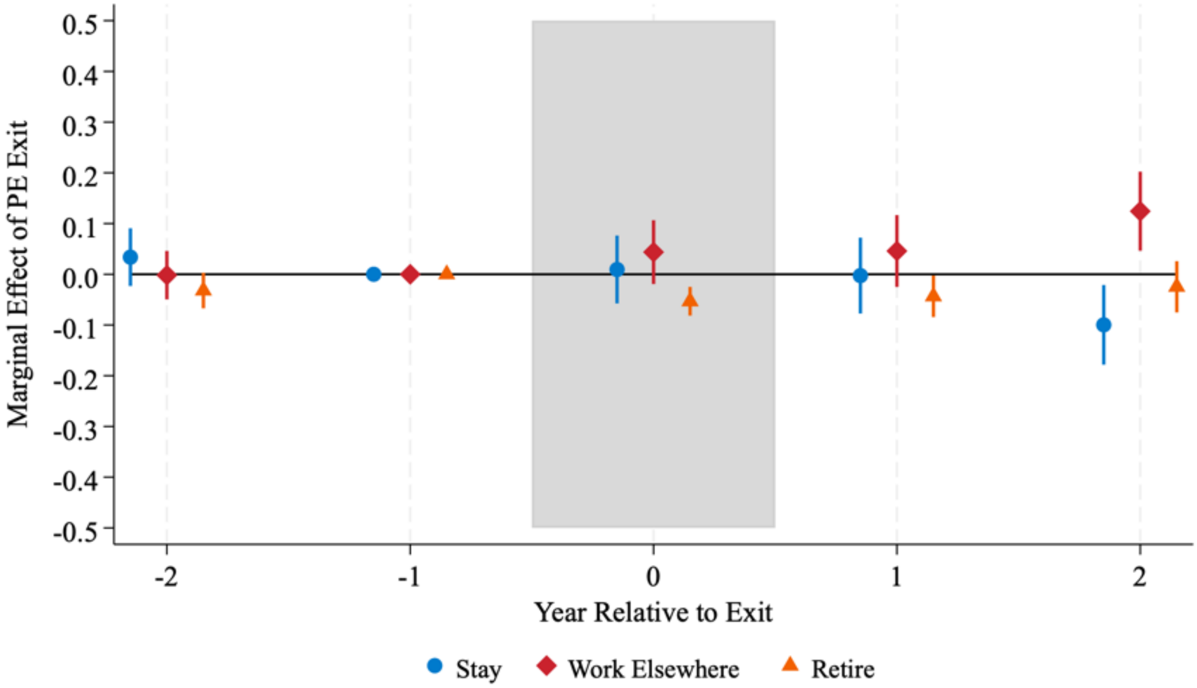


**Panel d) Difference: Family medicine – all other specialties**

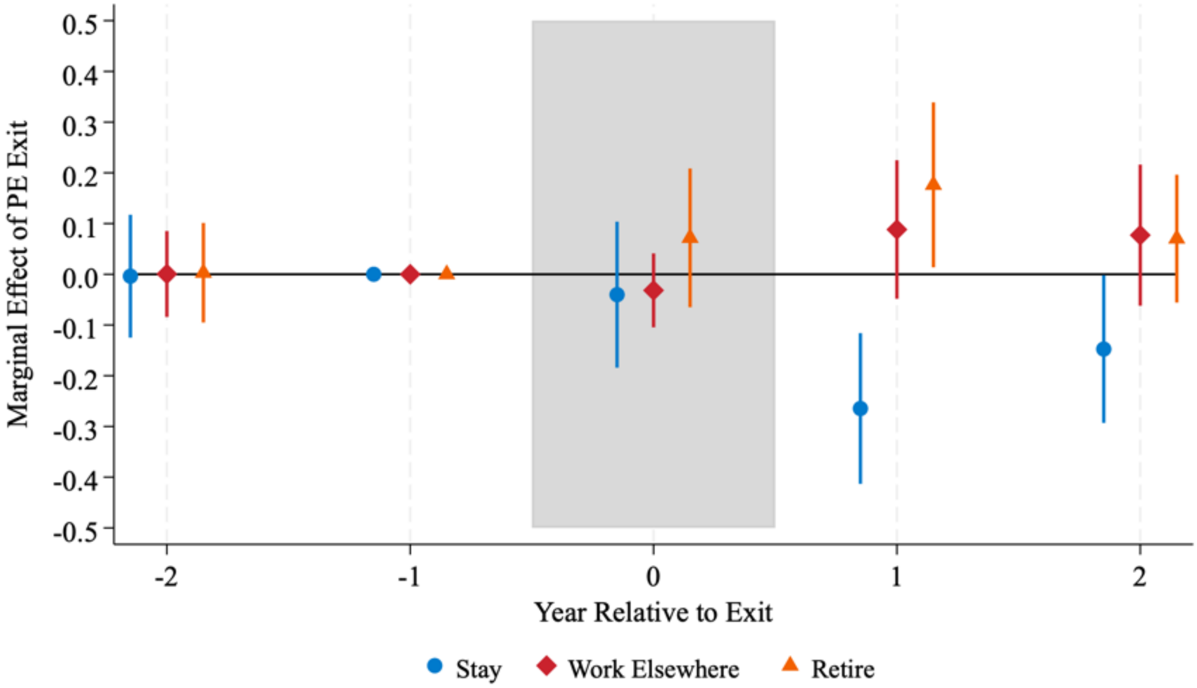


© 2025 Berquist V et al. *JAMA Health Forum*.

**Panel e) Difference: Dermatology – all other specialties**

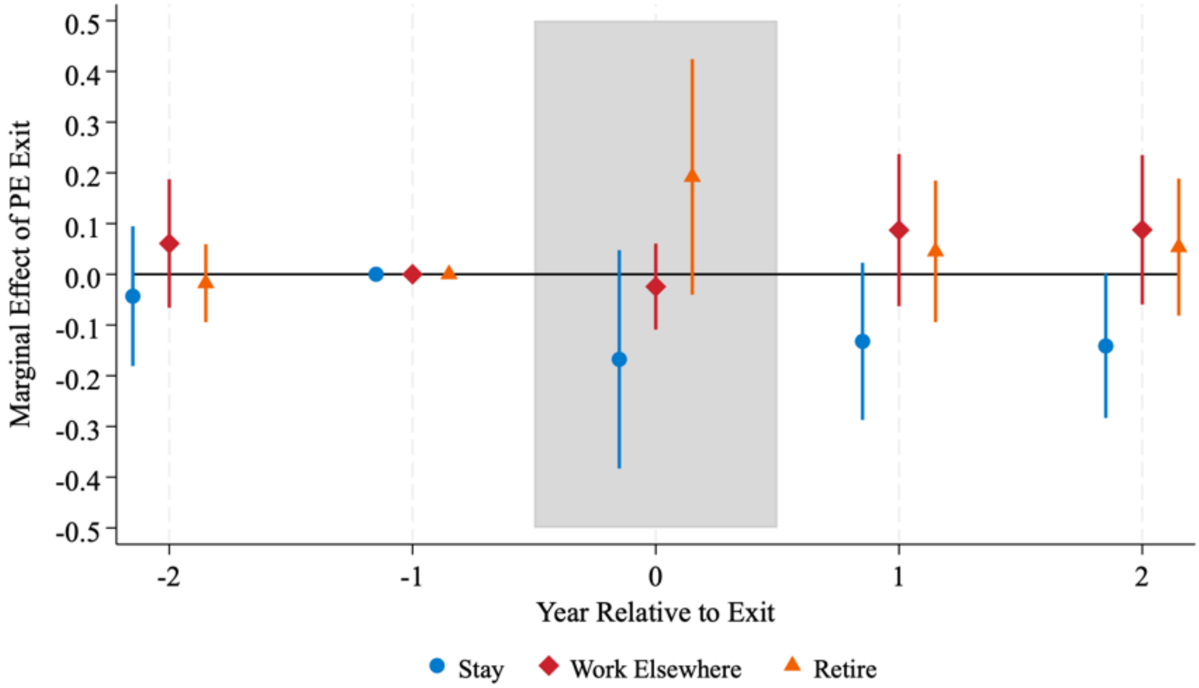


Notes: The figure plots the marginal effects from multinomial logit specifications for physician employment decisions in which the indicators for years relative to PE exit are interacted with indicators reflecting physician specialty. The data is at the physician-year level and point estimates for stay and retire are offset on the x-axis slightly for readability. The points represent the corresponding coefficients, and the vertical bars are 95% confidence intervals. Panels (a), (b), and (c) plot the effects separately by specialty; panels (d) and (e) plot the difference between these groups to illustrate whether they are statistically significant. Panels (a), (b), and (c) are estimated using a single, pooled multinomial logit regression where a PE exit indicator for each year relative to exit is interacted separately with an indicator which equals one if the physician's specialty is family medicine (coefficient estimates reported in panel (a)), an indicator which equals one if the physician's specialty is dermatology (coefficient estimates reported in panel (b)), and an indicator which equals one if the physician's specialty is neither family medicine nor dermatology (coefficient estimates reported in panel (c)). Panels (d) and (e) plot the interaction terms of a single, pooled multinomial logit regression with indicators for PE exit in each year relative to exit and indicators for PE exit in each year relative to exit interacted with indicators for family medicine and dermatology. Both regressions control for graduation year bins and indicators for family medicine, dermatology, and all other specialties interacted with year relative to exit. The shaded region indicates the year of PE exit. Standard errors are clustered at the physician level.

© 2025 Berquist V et al. *JAMA Health Forum*.
