# Supplementary material for: Sale of Private Equity–Owned Physician Practices and Physician Turnover
Source: JAMA Health Forum. 2025 Feb 14;6(2):e245376. doi: 10.1001/jamahealthforum.2024.5376 (PMC11829224; doi:10.1001/jamahealthforum.2024.5376)
Supplement: Supplement 2. — Data Sharing Statement [file jamahealthforum-e245376-s002.pdf]

Data Sharing Statement

Berquist. Sale of Private Equity–Owned Physician Practices and Physician Turnover. *JAMA Health Forum*. Published February 14, 2025. doi:10.1001/jamahealthforum.2024.5376

Data
Data available: Yes
Data types: Participant data with identifiers
How to access data: toriberquist@gmail.com
When available: With publication

Supporting Documents
Document types: None

Additional Information
Who can access the data: All data can be shared provided the requester has a license to access Pitchbook.
Types of analyses: Any purposes
Mechanisms of data availability: with written confirmation of a Pitchbook license
